# Maternal and fetal cytomegalovirus infection: diagnosis, management, and prevention

**DOI:** 10.12688/f1000research.12517.1

**Published:** 2018-03-01

**Authors:** Robert F. Pass, Ravit Arav-Boger

**Affiliations:** 1 Department of Pediatrics, University of Alabama at Birmingham, Children’s of Alabama, 1600 7th Avenue South, Suite 108, Birmingham, AL, 35233, USA; 2 Department of Pediatrics, Division of Infectious Diseases, Johns Hopkins University School of Medicine, Baltimore, MD, 21287, USA

**Keywords:** Cytomegalovirus, Prenatal infection, Fetal infection, Congenital infection, Fetal diagnosis, CMV prevention, CMV hyperimmune globulin, Valacyclovir

## Abstract

Congenital cytomegalovirus infection is a major cause of central nervous system and sensory impairments that affect cognition, motor function, hearing, language development, vestibular function, and vision. Although the importance of congenital cytomegalovirus infection is readily evident, the vast majority of maternal and fetal infections are not identified, even in developed countries. Multiple studies of prenatal cytomegalovirus infections have produced a body of knowledge that can inform the clinical approach to suspected or proven maternal and fetal infection. Reliable diagnosis of cytomegalovirus infection during pregnancy and accurate diagnosis of fetal infection are a reality. Approaches to preventing the transmission of cytomegalovirus from mother to fetus and to the treatment of fetal infection are being studied. There is evidence that public health approaches based on hygiene can dramatically reduce the rate of primary maternal cytomegalovirus infections during pregnancy. This review will consider the epidemiology of congenital cytomegalovirus infection, the diagnosis and management of primary infection during pregnancy, and approaches to preventing maternal infection.

## Introduction

Surveys show that very few women of childbearing age in North America have heard of congenital cytomegalovirus (CMV) infection, are aware of the importance of CMV as a cause of birth defects, or know the likely sources of maternal CMV infection or possible means of prevention ^1– 4^. Similar conclusions were reached on the basis of results from over 10,000 respondents to a web-based survey of students, administration, and faculty at an Italian university ^5^. A survey of 305 practicing obstetricians from across the US found that fewer than half counseled patients on the prevention of CMV infection ^6^; studies in France and the Netherlands also reported significant gaps in knowledge of maternal and congenital CMV infection among providers of prenatal care ^7, 8^. There are many published studies of CMV infection in pregnant women; the dangers of maternal CMV infection and the importance of congenital infection as a cause of disability are well known to clinical investigators in this field and to many health-care professionals. However, clinicians and public health officials struggle to translate knowledge gained from studies into messages and actions that can prevent maternal and congenital CMV infections.

Congenital CMV infection is common, occurring in approximately 0.5% to 1% of live births in the US and Europe; higher rates are observed in low-income populations ( Table 1) ^9– 20^. A systematic review of birth prevalence of congenital CMV in developing countries included 11 studies with sample sizes ranging from 317 to 12,195 and reported rates from 0.6% to 6.1% ^21^.

**Table 1.** Rates of congenital cytomegalovirus (CMV) infection from selected studies that screened newborns using virus culture or polymerase chain reaction to detect CMV in saliva, urine, or dried blood spots.

Study	Socioeconomic status	Method	Number	CMV infections, number (percentage)
Hamilton, ON, Canada, 1973–1976 ^14^	Mixed	Urine, VC	15,212	64 (0.42)
Malmo, Sweden, 1977–1986 ^9^	Mixed	Urine, VC	16,474	76 (0.46)
Sapporo, Japan, 1977–2002 ^17^	Mixed	Urine, VC	11,938	37 (0.30)
Birmingham, USA, 1978–1984 ^20^	Middle/upper	Urine, VC	8,545	47 (0.55)
London, UK, 1979–1982 ^18^	Mixed	Saliva, VC	14,200	42 (0.30)
Birmingham, USA, 1980–1990 ^13^	Lower	Urine, VC	17,163	215 (1.25)
Brussels, Belgium, 1996–1998 ^12^	Mixed	Urine, VC	3,075	15 (0.49)
Sao Paulo, Brazil, 2003–2007 ^16^	Lower	Urine or saliva, PCR	8,047	87 (1.08)
Eight US cities, 2008–2009 ^11^	Mixed	Saliva, PCR and rapid VC	34,989	177 (0.51)
Israel, 2011–2012 ^10^	Mixed	Saliva, PCR	9,845	48 (0.57)
Turkey, 2013–2014 ^19^	Mixed	Saliva, PCR	944	18 (1.91)
Paris, 2013–2015 ^15^	Mixed	Saliva, PCR	11,715	44 (0.37)

PCR, polymerase chain reaction; VC, virus culture.

Congenital CMV infection is a leading cause of hearing and central nervous system (CNS) impairments in children. Although the majority of infants with congenital CMV infection are normal at birth and survive without sequelae, around 15% will have damage to hearing, vision, cognition, or motor function with wide variation in the severity of impairments ^22^. The number of infants with disabilities due to congenital CMV infection born each year in the US has been estimated at around 6,000 ^23^. The economic significance of congenital CMV infection in the US was estimated in 1992 at around $1.86 billion per year ^24^. The negative impact on the quality of life of patients assessed at school age and of their parents is substantial ^25^.

## Epidemiology of maternal and congenital cytomegalovirus infection

Factors that influence the transmission of CMV are those associated with person-to-person contact with body fluids. Following initial infection, CMV is shed in multiple body fluids, including saliva, urine, semen, cervical/vaginal secretions, and breast milk, for months to years. The transmission of CMV through breast milk and, to a lesser extent, during birth is common. In populations with high prevalence of maternal infection and high rates of breastfeeding, the majority of infants acquire CMV by 1 year of age ^26^. Infants shed CMV in body fluids for years and frequently transmit CMV to those with whom they have close contact: other infants, toddlers, and children and parents and other caregivers ^27, 28^. In some developing countries, the prevalence of CMV infection reaches 100% in early childhood ^26^. In developed countries, the prevalence of CMV infection increases with age well into adulthood, and it is common for a high proportion, even the majority, of a population to reach adulthood without acquiring CMV ^29^. Contact with young children of preschool age, especially those who attend a day-care center and are younger than 3 years of age, carries a high risk of infection. Very high rates of CMV infection have been reported in children in day-care centers; in some centers, the majority of children between 1 and 2 years of age are shedding CMV ^27^. The risk of CMV infection is particularly relevant for mothers of children attending day-care and for child-care workers, many of whom are women of childbearing age. Rates of incident CMV infection 5- to 10-fold higher than the expected rate of 2% per year in the general population have been reported for day-care workers ^30, 31^. Incidence rates of around 50% per year have been observed for CMV-seronegative mothers whose infant or toddler is known to be shedding CMV ^32, 33^. Sexual activity is also an important means of transmitting CMV. In sexually active adolescents and in adults attending sexually transmitted disease clinics, rates of CMV infection of around 35% per year have been reported ^34, 35^. Rates of incident CMV infection related to exposure to young children and to sexual activity are listed in Table 2 ^30, 31, 33, 34, 36– 39^.

**Table 2.** Reported high rates of cytomegalovirus infection among adolescents and adults.

Study	Exposure	Rate, percentage per year
Mothers ^39^	Premature infant with transfusion- acquired cytomegalovirus	47
Parents ^33^	Child in group day-care	21
	Child attends day-care and is shedding cytomegalovirus	30
Day-care workers, Iowa ^37^	Caregiver for preschool-age children	7.9
Day-care workers, Richmond, VA ^30^	Caregiver for preschool-age children	11
Day-care workers, Toronto ^36^	Caregiver for preschool-age children	12.5
Day-care workers, Birmingham, AL ^31^	Caregiver for preschool-age children	20
Adolescents, aged 12–15, Cincinnati, OH ^38^	Urban adolescent clinic	13.8
Women, Seattle, WA ^34^	Clients of sexually transmitted disease clinic	37

The risk of transmission of CMV to the fetus after primary maternal infection is often estimated to be around 35%; however, the rate can range from roughly 20% to 75% of pregnancies ^26^. Dating the onset of maternal CMV infection is difficult because it is unusual for symptoms suggestive of CMV to occur and to be recognized as such. Much of the available information on the timing of maternal infection is based on methods that cannot precisely assign maternal infection to immediately preconception or the first, second, or third trimester. Because maternal CMV infection is active for months, it is also possible that transmission to the fetus occurs weeks after the onset of maternal infection. As has been seen with other congenital infections, maternal CMV infection early in pregnancy is less likely to be transmitted to the fetus (around 20%), but the infected fetus is more likely to be symptomatic at birth and to have disabilities than with infection later in gestation ^40– 43^. With maternal infection in the third trimester, the rate of transmission to the fetus is approximately 75% and disease is uncommon and less severe when it occurs ^44^. CMV can also be transmitted from mother to fetus if the mother had CMV infection in the past and was immune at the time of conception. These have been referred to as non-primary, secondary, or recurrent maternal infections, and the last of these terms reflects uncertainty as to whether they are the result of reactivation of latent virus, reinfection, or persistent active infection from a relatively recent primary infection. Evidence suggests that these non-primary maternal infections that are transmitted to the fetus are the result of reinfection with a virus that varies from the previous strains in immunologically important epitopes on envelope glycoproteins ^45, 46^. Rates of congenital CMV infection due to non-primary maternal infection vary widely among populations, ranging from approximately 0.1% to 1% or higher; they are generally higher in developing countries and in populations in which the overall prevalence of CMV infection is high, and they are less common in middle- and upper-income populations in developed countries ^20, 47– 49^. Estimating the rate of non-primary congenital CMV infection is possible when data on age-related prevalence of infection show that near-universal infection occurs during childhood or when the congenital infection rate is measured in a population sample known to be seropositive before conception. It is uncommon for prenatal CMV serology to be available, and some studies have classified CMV antibody-positive patients as non-primary infections on the basis of lack of IgM antibody and presence of high-avidity IgG antibody. The reliability of this approach has been questioned ^50^. There is variability among patients in the persistence of CMV-specific IgM antibody and maturation of IgG antibody response to high avidity. In addition, there is variability among laboratory assays for IgM antibody and IgG antibody avidity. Although most non-primary maternal infections do not result in transmission to the fetus, there is no screening test for non-primary infection during pregnancy that will predict the likelihood of congenital infection. The diagnosis and management of CMV infection during pregnancy have therefore focused on primary maternal infection.

## Pathophysiology of maternal and fetal cytomegalovirus infection

Although some clinical factors that contribute to the transmission of CMV from mother to fetus and to the occurrence of fetal disease are known, understanding of the pathophysiologic mechanisms that affect transplacental transmission of virus and virulence of fetal infection is limited. The role of gestational age at the time of maternal infection is clearly an important determinant of both transmission and outcome, as noted above. The presence of maternal immunity from past CMV infection decreases the risk of fetal infection by around 70% ^47^. Virus inoculum at the time of maternal infection could be important, but it has not been possible to investigate this. Cytokine profiles in amniotic fluid have been studied in pregnancies in CMV-seropositive women, comparing results according to whether or not the fetus was infected ^51^. A number of cytokines, including pro- and anti-inflammatory markers, were upregulated in amniotic fluid from women who transmitted virus to the fetus. Elevated levels of tumor necrosis factor-alpha and chemokine ligand 2 (CCL2), a recruiter of monocytes, memory T cells, and dendritic cells to sites of inflammation, have been found in CMV-infected placentas from stillborn babies compared with uninfected placentas and those infected with other viruses or bacteria ^52^. Whether these cytokine and chemokine changes at the maternal–fetal interface facilitate transplacental transmission of virus or result from fetal infection is unclear. Viral genotypes have been investigated in relation to transmissibility, but it appears that all strains are transmissible ^53^.

Recent publications point to host genetic factors that influence innate immune responses as potentially important determinants of maternal CMV infection, transplacental transmission of virus, and fetal disease. Toll-like receptors (TLRs) are membrane-spanning proteins that activate immune responses upon recognition of microbial proteins and are a key element of innate immune response. A study of pregnant women with CMV infection and age-matched controls examined TLR 2, 4, and 9 genotypes and found that single-nucleotide polymorphisms (SNPs) in TLR 9 were associated with decreased occurrence of CMV infection ^54^. The same laboratory examined a number of pro-inflammatory cytokine genes in 20 fetuses and newborns with congenital CMV infection and 31 uninfected controls and found that specific SNPs in *IL1A* and *IL1B* were associated with the risk of congenital infection as well as the risk of symptomatic disease ^51^. These findings will require replicate studies. Nucleotide-binding oligomerization domains 1 and 2 ( *NOD1* and *2*) are genes that encode cytoplasmic proteins that serve as pattern recognition receptors that initiate innate immune responses when stimulated by peptidoglycan moieties of Gram-positive and Gram-negative bacteria. With their cytoplasmic location, NOD proteins are well positioned to react to viral infection ^55^. NOD1 and NOD2 are activated by CMV and trigger innate immune responses which suppress CMV infection ^56, 57^. A recent study found that SNPs in *NOD1* highly correlate with risk of CMV acquisition in young women ^56^. This study used samples from young women who participated in a CMV vaccine trial, were seronegative at enrollment, and were followed for 3.5 years for evidence of CMV infection ^58^. Of 768 selected SNPs in 29 innate immune response genes, six SNPs were significantly different between CMV-infected and non-infected women, three of which were in the *NOD1* gene. NOD1 and NOD2 are particularly attractive for further study in relation to maternal and fetal CMV infection because of their presence at the maternal–fetal interface. NOD1 is expressed in term placenta, and both NOD1 and NOD2 are expressed by syncytiotrophoblast and cytotrophoblast cells in first-trimester placental villi ^59– 61^. The expression of these intracellular receptors by the syncytiotrophoblast cell layer is notable because these cells lack TLRs ^62^.

## Identification and management of maternal and fetal infection

Primary maternal CMV infections during pregnancy are usually not recognized clinically. A study that screened large numbers of pregnant women for CMV antibody and followed those who were seronegative through seroconversion reported a febrile illness with signs and symptoms possibly attributable to CMV in around 10% of those who seroconverted ^20^. Although a mononucleosis-like illness with prolonged fever, malaise, adenopathy, skin rash, pharyngitis, and abnormal hepatic transaminases can be seen with primary CMV infection, it is uncommon even in pregnant women. No symptom complex has been associated with recurrent or non-primary CMV infection in pregnant women. Decision making regarding the management of CMV infections in pregnant women is necessary when evaluation of a patient with a febrile illness establishes primary CMV infection; when a patient with high risk for CMV infection requests evaluation or the prenatal care provider recommends it; and when an obstetric practice identifies a primary maternal infection through screening. These situations are the exception, and it is important to acknowledge that the vast majority of maternal CMV infections during pregnancy are not recognized and therefore escape any management decisions.

## Patient engagement

When primary CMV infection is suspected in a pregnant woman, it is essential to engage the patient in decision making; many patients will want their partner or another support person to be present in key discussions. The patient must be provided with basic information on the risk of transmission of virus to the fetus and the risk of damage if the fetus is infected. The patient will also need to know how she will be evaluated to confirm recent primary infection, including information on any risk to mother or fetus from testing and the positive and negative predictive value of test results. Risks of invasive prenatal testing procedures for fetal infection can include miscarriage, although the risk is low for the most widely used diagnostic procedure: amniocentesis (0.11% pooled procedure-related risk from meta-analysis including 42,716 women) ^63^. If fetal infection is proven, what subsequent steps will be acceptable to the patient? The choices are limited: termination of pregnancy and treatments of unproven efficacy and potential risk (antiviral or CMV immune globulin). After receiving the best information available on the risks and benefits of fetal testing and available interventions, some patients will prefer to avoid further testing. In those cases, careful assessment of the newborn for signs of congenital infection and testing for congenital CMV infection can be of value in identifying newborns who merit antiviral treatment or in anticipating possible sequelae.

## Laboratory confirmation of recent maternal cytomegalovirus infection

If the patient with a possible primary CMV infection during pregnancy wishes to proceed, the first step is to pursue laboratory confirmation with the goal of determining whether recent primary CMV infection is present. Conversion of CMV IgG antibody from negative to positive is the most straightforward confirmation but is possible only if there are paired serum samples that can pinpoint infection during pregnancy, an unlikely scenario. If the patient is positive for both IgG and IgM antibody to CMV when first tested, further evaluation is required. CMV IgM antibody does not reliably identify primary CMV infection during pregnancy, and this is due to variability in the persistence or appearance of IgM antibody after primary infection and to variable sensitivity and specificity of IgM antibody tests for the detection of recent primary infection. Testing for IgM antibody to specific viral proteins has been used to enhance the specificity of CMV IgM results, but such testing is unlikely to be widely available ^64, 65^. If previously collected serum and results from prior CMV serologic testing are not available, the next step is testing for avidity of CMV IgG antibody. With primary infection, the initial IgG is of low avidity, maturing to high avidity within a few months or longer ^66^. The presence of IgM antibody and low-avidity IgG antibody provides strong evidence of recent primary infection. CMV IgG avidity test results can be misleading (low IgG avidity when infection is not recent) when used on sera that lack CMV IgM antibody and have low levels of IgG antibody ^67, 68^. Proprietary kits for CMV IgG and IgM antibodies and for avidity testing are available, and many clinical laboratories offer these tests. However, there is variability in the performance of commercial kits, and sera that meet criteria for recent primary CMV infection on the basis of results with one commercial assay may not meet the criteria of a different commercial assay ^67– 70^. It is important for the clinician evaluating a woman with possible primary CMV infection to understand the limitations of the available laboratory tests for determining the timing of recent CMV infection.

Primary CMV infections result in shedding of virus in multiple body fluids, which can persist for months and recur years after primary infection. Neither maternal shedding of CMV in urine or saliva nor CMV DNAemia proves primary infection during pregnancy ^71^. A study that detected primary infection in pregnant women on the basis of CMV IgM antibody and low-avidity IgG antibody found that the risk of fetal infection was higher when viremia was detected by polymerase chain reaction (PCR) in a first-trimester maternal serum sample ^72^. A study of blood collected at the time of prenatal amniocentesis of 239 women with primary CMV infection reported that the risk of congenital CMV infection was increased threefold when maternal CMV DNAemia was detected by PCR ^73^. Maternal viremia in women with proven primary CMV infection is associated with fetal infection, but fetal diagnosis relies on amniocentesis.

## Evaluation of the fetus

A positive PCR or virus culture result from amniotic fluid is considered proof of fetal infection ^74^. A negative result is reassuring but may not rule out fetal infection. Amniotic fluid collected prior to 20 weeks of gestation or less than 6 weeks from onset of maternal infection can be negative and fetal or congenital infection subsequently proven ^75– 77^. A retrospective study reported that infants who were proven to have congenital CMV infection after a negative test of amniotic fluid were less likely to have significant clinical abnormalities at birth and none (0 out of 47) had long-term sequelae ^78^. This is not surprising, as these congenital infections are transmitted later in pregnancy.

Cordocentesis has been used to quantitate CMV in fetal blood and to measure fetal platelet count and antibody response to infection. In two retrospective studies of pregnancies with confirmed maternal CMV infection, high viral load in fetal blood was a predictor of symptomatic fetal and congenital CMV infection, especially when maternal infection occurred in the first trimester ^79, 80^. In both of these reports, the rates of fetal or newborn CMV-related sequelae were higher than expected with primary maternal infection during pregnancy, and there were also high rates of termination of pregnancy. Retrospective studies of an invasive procedure are subject to confounding by indication; invasive procedures are more likely to be performed in patients with perceived greater risk of worse outcome. Zavattoni *et al*. noted that the risk of spontaneous abortion is increased with cordocentesis and recommended limiting cordocentesis at 20–21 weeks of gestation to pregnancies with CMV-infected fetuses at high risk of poor outcome with early gestation (≤8 weeks) infection ^80^. An informative study regarding risks attributable to cordocentesis included a large group of pregnancies being evaluated for fetal hemoglobinopathies or karyotype abnormalities ^81^. Risk of cordocentesis was determined by comparing fetal loss rates in 5,506 pregnancies that underwent cordocentesis but did not have hemoglobinopathy or karyotype abnormalities with a matched control group of 5,039 that did not have cordocentesis. The cordocentesis group had a fetal loss rate of 1.9% (relative risk 1.9, 95% confidence interval 1.4–2.7) compared with controls. Cordocentesis is not necessary for the diagnosis of fetal CMV infection, although it could contribute information of value in predicting the outcome of fetal infection.

Microcephaly, periventricular calcifications or echogenicity, parenchymal echogenic foci, ventricular dilatation, pseudocysts, abnormal gyral development, and hypoplastic corpus callosum have been detected by ultrasound imaging of fetuses with CMV infection ^82, 83^. Extracerebral signs of fetal infection include hyperechogenic bowel, enlargement of liver and spleen, ascites, hydrops, pericardial effusion, and placental enlargement ^82^. It is very common for pregnant women to have routine ultrasound screening for fetal well-being at around 18–20 weeks of gestation, and the finding of ultrasound abnormalities suggestive of congenital CMV infection can lead to serologic evaluation of the mother for primary CMV infection and diagnostic amniocentesis to test for fetal infection. The ultrasound abnormalities mentioned above are not specific for fetal CMV infection, but when fetal infection has been proven they can inform prognosis. In fetuses with proven CMV infection, both fetal thrombocytopenia and ultrasound abnormalities were associated with poor outcome (abnormal clinical exam or hearing loss at 6 months or severe disease on postmortem exam of aborted fetus) ^82^. A retrospective study of 600 women found to have primary CMV infection during pregnancy reported 51 with abnormal ultrasounds; 23 out of 51 fetuses had congenital CMV infection ^84^. The predictive value of ultrasound abnormalities for the occurrence of congenital infection was only 45%. Fetal ultrasound abnormalities often associated with CMV infection (choroid plexus cysts, hyperechogenic bowel, ventriculomegaly, and intrauterine growth retardation) were seen in a number of pregnancies that did not result in congenital CMV infection. Limiting analysis to 154 pregnancies in which fetal CMV infection was proven, this study found that ultrasound abnormalities were predictive of symptomatic infection (one or more clinical or laboratory abnormalities associated with congenital CMV infection) in the newborn with 78% positive predictive value. However, midgestation ultrasounds of infected fetuses were often normal when subsequent ultrasound or newborn exam revealed evidence of disease.

When fetal infection has been proven by the detection of CMV in amniotic fluid and ultrasound shows clear evidence of damage to the CNS, a poor outcome (severely symptomatic newborn who will have sensory, cognitive, or motor disabilities or even fetal or neonatal death) is very likely. In situations in which fetal damage is less severe or not detected by prenatal ultrasound, information on the risk of having a symptomatic newborn with CNS disabilities is desirable. A study aimed at identifying potential fetal cord blood prognostic markers of symptomatic congenital CMV infection found that β2-microglobulin, platelet count, IgM antibody to CMV, and CMV DNAemia were predictive, especially in combination ^85^. A study of women with primary CMV infection found that maternal CMV DNAemia alone at the time of amniocentesis did not reliably predict symptomatic congenital infection ^73^. A review of fetal CMV infections that did not have severe brain ultrasound abnormalities at the time of diagnostic amniocentesis reported that the combination of non-severe ultrasound abnormalities, elevated viral load in amniotic fluid, low fetal platelet count, and high level of fetal CMV DNAemia was strongly associated with neonatal symptomatic congenital CMV infection ^79^. Further research is needed on approaches to the identification of CMV-infected fetuses who will have no sequelae as well as those who are likely to have significant CNS sequelae.

## Prenatal screening for maternal cytomegalovirus infection

Screening of pregnant women for CMV infection has been a key component of studies of maternal and fetal infection and is being provided as a clinical service in some obstetric clinics. Whether pregnant women should routinely undergo prenatal laboratory screening for CMV infection is a controversial issue. The vast majority of maternal CMV infections are clinically silent. Universal screening at the first prenatal visit could dramatically increase the number of primary maternal CMV infections identified. However, the screened population would be quite different from women who have been studied because of suspected CMV infection in terms of the pretest probability of maternal infection. The ratio of false positives to true positives would be much higher in the screened population than in women preselected for suspected CMV infection, and many women would undergo unnecessary additional testing and possibly invasive procedures. No known guidelines, professional societies, or public health policies currently recommend universal maternal prenatal screening for primary CMV infection. The development of accurate, standardized, and widely available assays for CMV IgM antibody and IgG avidity could change the risk–benefit ratio for maternal screening for CMV. The development of interventions of proven effectiveness for preventing the transmission of CMV from mother to fetus or for the treatment of fetal infection will create a need for universal prenatal screening. A recent review discusses issues related to universal prenatal screening for primary maternal CMV infection ^86^.

## Intervention to prevent transmission of cytomegalovirus to the fetus

Administration of CMV hyperimmune globulin (CMV HIG) during pregnancy has been evaluated for the prevention of transmission of CMV from mother to fetus; results on efficacy have been mixed. A 2005 study suggested that prenatal administration of CMV immune globulin could prevent transmission of the virus to the fetus ^87^. Newborns of women who did not have amniocentesis were tested for congenital CMV infection at birth, and the rates of infection were compared according to whether or not the mother received CMV HIG during pregnancy. Congenital CMV infection was found in 6 (16%) out of 37 newborns of treated mothers compared with 19 (40%) out of 47 newborns from the untreated group, a difference that was statistically significant. In this study, maternal infection occurred earlier in the treatment group, and this could be a source of bias, as there is evidence that transmission of virus to the fetus is less likely with first-trimester infection than with later gestational infection ^40, 44^. Without randomization, a control group, and masking, it is very difficult to convincingly demonstrate efficacy. This is particularly true when examining the impact of prenatal treatment on CMV infection and disease in the fetus or newborn. The outcome of prenatal infection is highly variable, and the means of detecting primary maternal infection vary in sensitivity and specificity. This study, like other retrospective observational studies of CMV HIG, is subject to biases, such as allocation bias as well as confounding ^88, 89^.

Although these non-randomized studies do not establish efficacy of CMV HIG for the prevention of mother-to-fetus transmission of CMV, they are important because they stirred interest in conducting the randomized controlled clinical trials which have followed. In addition to the controversy over efficacy, dosing interval, and safety of this intervention, there is evidence based on laboratory comparison of CMV-specific antibody levels and virus-neutralizing activity of commercial products that suggests that standard intravenous immune globulin preparations could be as effective as the much more expensive HIG preparations ^90^.

A randomized, placebo-controlled, observer- and participant-blind clinical trial compared CMV HIG in women with primary CMV infection during pregnancy with a saline placebo ^91^. Subjects were women 18 years or older, between 5 and 26 weeks of gestation, and within 6 weeks of the estimated onset of CMV infection. They received study treatments monthly up to 36 weeks of gestation. In the intent-to-treat analysis (all enrolled subjects), congenital CMV infection was found in 18 (30%) out of 61 recipients of CMV HIG and in 27 (44%) out of 62 placebo recipients, a difference that was not statistically significant ( *p*=0.13). Obstetrical adverse events were more frequent in the CMV HIG group (13%) than in the placebo group (2%) ( *p*=0.06). There was no difference in the clinical spectrum/outcome of congenital CMV infection between treatment groups. A similar number of women in each randomization group underwent prenatal amniocentesis, and fetal infection was identified in 8 out of 33 in the CMV HIG group and in 10 out of 26 in the placebo group, not a statistically significant difference. When only pregnancies in which the fetus was known to be uninfected at initiation of treatment were considered, the congenital CMV infection rates were 3 out of 25 in the CMV HIG group and 0 out of 15 in the placebo group. Although the results of this clinical trial do not support the use of CMV HIG for either the prevention of CMV transmission from mother to fetus or the treatment of fetal infection, the relatively small sample size does not exclude a benefit. A larger, multi-institution randomized, placebo-controlled, double-blind clinical trial is under way in the US with an expected date of completion in late 2018 ^92^. More complete knowledge of the kinetics and maturation (avidity and epitope specificity) of the maternal antibody response to primary CMV infection during pregnancy along with improved understanding of the effective dose and pharmacokinetics of immune globulin (or perhaps monoclonal antibody preparations) could lead to advances in the use of passive immunization to prevent fetal infection.

## Intervention to treat fetal cytomegalovirus infection

The outcome of congenital CMV infection is measured by the occurrence of CNS and sensory (hearing and vision) sequelae and is highly variable. Among congenitally infected newborns, around 90% have no signs or symptoms of congenital infection at birth and around 5–15% of these asymptomatic newborns have sequelae, almost entirely hearing loss. Among the 10% who are symptomatic at birth, sequelae (singly or in combination), including hearing loss, vision impairment, mental retardation, or cerebral palsy, are much more frequent. Follow-up studies that are not biased by oversampling of referral patients report that roughly 50% of congenitally infected newborns who are symptomatic will have sequelae ^93, 94^. Those with moderate or severe outcomes will usually be identified by 1 year of age. Given the natural history of congenital CMV infection, adequate assessment of potential therapies requires a randomized clinical trial. The efficacy of ganciclovir treatment of newborns with symptomatic congenital CMV infection was established by a randomized clinical trial comparing 6 weeks of intravenous ganciclovir with no treatment ^95^. Because it was not considered reasonable to give newborns 6 weeks of intravenous placebo, a placebo was not used. No randomized, placebo-controlled clinical trials have established the efficacy of potential prenatal treatments for fetal CMV infection, but valacyclovir and CMV immune globulin have been studied in pregnant women, with the goal of treating fetal infection.

A 2005 study (referenced above in relation to the prevention of fetal infection) reported treatment of fetal infection with CMV HIG in pregnancies complicated by a primary maternal CMV infection with amniotic fluid or fetal blood positive for CMV ^87^. The outcome of fetal infection was compared according to whether or not the mother accepted or declined prenatal treatment with CMV HIG. The first dose of HIG was given at a median of 24 weeks of gestation (interquartile range 22–27 weeks) intravenously, into amniotic fluid, or intravenously to the fetus via umbilical vein. Only 1 of 15 babies from the treatment group had CMV-related sequelae, whereas 5 of 7 babies who did not receive prenatal CMV HIG had severe CNS sequelae. Six newborns from the treated group did not have congenital CMV infection when tested at birth, raising concern about misclassification of fetal infections. The rate of severe CNS sequelae in the untreated group was much higher than expected from many published studies of the outcome of congenital CMV infection. Retrospective studies that reported infants’ outcome (disease attributable to congenital CMV infection) when the mother received CMV HIG during pregnancy have suggested benefit, but variability in the criteria for confirming primary maternal CMV infection, variation in the preparation and administration of CMV HIG, and selection bias make it difficult to draw firm conclusions ^88, 89, 96^. A randomized clinical trial of CMV HIG found no difference in the severity of congenital CMV infection between babies of mothers who received CMV HIG and those who received placebo ^91^.

A phase II open-label study of prenatal treatment of congenital CMV infection with oral valacyclovir reported advantageous biological effects and improved outcome compared with historical controls ^97^. The positive biological effects were a statistically significant decrease in fetal blood viral load and increase in fetal platelet count. The study population was pregnant women with CMV PCR-positive amniotic fluid at more than 21 weeks of gestation with evidence of fetal CMV disease based on extracerebral ultrasound findings, fetal platelet counts or fetal blood viral load, or mild cerebral ultrasound abnormalities. The presence of severe cerebral ultrasound abnormalities and the absence of evidence of fetal disease were exclusions. The primary endpoint was the proportion of asymptomatic infants born to study participants. All participants received valacyclovir 2 g four times per day from enrollment until delivery or 24 weeks of treatment, whichever came first. Asymptomatic neonates were born to 34 (83%) out of 43 participants, compared with the rate of 43% in historical controls (derived from three published reports), and there was no overlap in 95% confidence intervals. The authors concluded that maternal treatment with valacyclovir during pregnancy had a beneficial effect on outcome of fetal CMV infection, and they suggested that a randomized trial comparing valacyclovir treatment to usual care could be used to confirm this effect.

There are currently no US Food and Drug Administration-approved treatments for fetal CMV infection, and the published results from studies of CMV HIG and antivirals in pregnant women with primary CMV infection fall well short of the type of evidence that will be required for such approval. Physicians who manage these patients are left to assess the available evidence and decide whether to offer off-label treatment with CMV HIG or valacyclovir. If treatment is recommended to a patient, it should be accompanied by forthright explanation regarding the level of certainty (or uncertainty) of benefit or adverse effects. Some experts recommend that such interventions be confined to clinical trials ^98^.

## Prevention of maternal cytomegalovirus infection

Preventing CMV infections in pregnant women is an important public health goal and was recognized as such in an Institute of Medicine analysis of vaccine priorities for the US ^99^. However, more than 15 years after that report was published, a licensed CMV vaccine appears to be years away. Preventing maternal infections by limiting exposure will be difficult because increased risk for CMV infections comes from two activities that are part of the social and family life of women of childbearing age: caring for young children and intimate contact with a spouse or partner. The Centers for Disease Control and Prevention in the US provide the following information on the prevention of CMV infection in pregnancy ( https://www.cdc.gov/features/prenatalinfections/index.html, accessed on 13 June 2017):

“CMV is passed from infected people to others through body fluids, such as saliva, urine, blood, vaginal secretions, and semen. Infants and young children are more likely to shed CMV in their saliva and urine than older children and adults. For pregnant women, the two most common ways they are exposed to CMV is through contact with saliva and urine of children with CMV infection and sexual activity. Regular hand washing, particularly after changing diapers, is a commonly recommended step to decrease the spread of infections and may reduce exposures to CMV”.

Whether this general advice is effective is unknown. Studies of hygiene education to decrease maternal CMV infection rates during pregnancy have had promising results. A 2004 study of women who had a child in day-care and who were pregnant or attempting pregnancy randomly assigned (by day-care center) 166 seronegative participants either to an intervention group that was instructed in prevention (handwashing, glove use, and education on risky behaviors) and informed that they were CMV seronegative or to a control group (no hygiene instruction and no information on their CMV serologic status) ^100^. Neither group was told whether their child was shedding CMV. The seroconversion rate was the same in the two groups: 7.8%. However, there was a statistically significant decrease in CMV infection rate from 42% to 6% when women who were pregnant at enrollment were compared with those who were not, leading the authors to conclude that the intervention could be effective for women who know they are pregnant and know that they are seronegative. Another study of hygiene education took place in a French obstetric clinic in which all pregnant women were being given information on CMV infection and serological testing for CMV was offered ^101^. For those who were seronegative at 12 weeks of gestation, counseling on how to prevent CMV infection was offered to the patient and her partner and a repeat serological test was performed at 36 weeks. Primary CMV infection occurred in 0.42% of women between 0 and 12 weeks of gestation and in 0.19% between 12 and 36 weeks of gestation, evidence that the counseling had reduced the CMV infection rate from 1.6% to 0.41% per year. A study in two Italian obstetric clinics was able to use sera obtained and stored at the time of aneuploidy testing at 11–12 weeks of gestation to identify and enroll 331 seronegative women for the intervention group ^102^. They were given information on the risks for and consequences of CMV infection in pregnancy as well as specific instructions (by video, in writing, and with pictorial teaching aids) on measures to prevent CMV infection; a brief review of information was presented at 18 weeks of gestation. The comparison group, enrolled at 36 weeks of gestation, did not receive the instruction on the prevention of CMV infection. Testing of archival sera and matching on maternal age, parity, and exposure to at least one risk factor provided a comparison group of 315. Congenital CMV infection, detected by PCR testing of amniotic fluid or newborn urine for CMV DNA, was found in 4 (1.2%) out of 331 in the intervention group compared with 24 (7.6%) out of 315 in the comparison group, a very meaningful decrease in congenital CMV infections ( *p*<0.001). These three studies provide convincing evidence that information on sources of CMV infection, risk to the fetus from maternal infection during pregnancy, and methods to decrease risk can prevent maternal and congenital CMV infections in pregnant women who are seronegative.

## Translating evidence into practice

“Expert clinicians, opinion leaders for congenital cytomegalovirus, researchers with expertise in congenital cytomegalovirus infection … from Europe, USA and Australia” met in a workshop in April 2015 and formulated consensus recommendations on the diagnosis, prevention, and treatment of maternal and congenital CMV infection ^103^. Their recommendations were published in June 2017 and can serve as a concise guide to decision making for clinicians providing care to mothers with CMV infection during pregnancy. Key recommendations from that report on the management of maternal infection are summarized in the Text box.

Recommendations for the management of maternal and fetal cytomegalovirus (CMV) infection, modified from those reported by Rawlinson *et al*. ^103^. Statements within quotation marks are as stated in the published report.

**Diagnosis**

- CMV serologic testing should be offered to a pregnant woman with an illness suggestive of primary CMV infection or when prenatal ultrasound (or magnetic resonance imaging) shows signs suggestive of fetal CMV infection.
- The diagnostic assessment for primary CMV infection should include CMV-specific IgG antibody in serum; a positive result from a woman known to be CMV antibody negative preconception or earlier in pregnancy is strong evidence of primary infection. If the first serum tested is CMV IgG positive, testing for CMV IgM antibody and for IgG antibody of low to moderate avidity is recommended to detect recent primary infection.
- Diagnosis of fetal infection is made by detection of CMV in amniotic fluid after 20–21 weeks of gestation and at least 6 weeks from the time of maternal infection. Real-time polymerase chain reaction is recommended for the detection of CMV in amniotic fluid.

**Prevention**

- All pregnant women should be provided information on congenital CMV infection. This information should include the potential dangers of CMV infection for the fetus, the most likely sources of infection, and steps to prevent infection.
- All health-care providers who serve young mothers or women of childbearing age should be informed about congenital CMV infection and prepared to provide information to women who are or intend to be pregnant.
- “Cytomegalovirus hyperimmunoglobulin should not be routinely administered to pregnant women with primary cytomegalovirus infection to prevent fetal cytomegalovirus infection.”
- “Routine antiviral therapy to prevent congenital cytomegalovirus infection during pregnancy is not recommended.”
- “Universal screening of all pregnant women to assist in the diagnosis of primary cytomegalovirus infection is currently not recommended.”

**Therapy**

- “Cytomegalovirus hyperimmunoglobulin treatment should not be routinely administered for fetal cytomegalovirus infection.”
- “Routine antiviral therapy to treat fetal cytomegalovirus infection during pregnancy is not recommended.”

The challenge to public health officials, obstetricians, and others involved in providing prenatal care or counseling is to incorporate knowledge of the risks for and consequences of maternal CMV infection into their practice with the goal of preventing CMV infection during pregnancy. This will require steps to identify women at increased risk of CMV infection and provide them with information on maternal and congenital CMV infection and instructions on practical steps that can be taken to decrease the risk of infection. Testing for primary CMV infection has a role when a prenatal patient has had an acute febrile illness not attributable to another specific etiology or when fetal ultrasound reveals findings suggestive of CMV infection or the patient is at higher risk because of contact with young children or sexual activity. However, one could argue that the prevention message should be provided to all prenatal patients because congenital infections that occur in newborns of mothers who are seropositive to CMV from past infection are likely reinfections from the same sources that are important for seronegative women. It is unlikely that practicing obstetricians and pediatricians or directors of child-care centers will be able to develop protocols for educating women on methods to reduce the risk of CMV infection without guidance from public health officials. National standards for education about and for the prevention of CMV infections in women who are pregnant or planning to become pregnant are needed, and this can be best accomplished by public health authorities responsible for disease prevention. The frequent, specific, and detailed guidance provided by the Centers for Disease Control and Prevention to obstetricians and pediatricians in 2016 and 2017 on the prevention of Zika virus infection during pregnancy and the evaluation of pregnant women or newborns with suspected Zika virus infection stands as an example. There is evidence that prevention efforts directed at pregnant women can reduce CMV infections by around 80%; it is time to translate that knowledge into medical practice standards and public health policy.
